# Deep learning supported machine vision system to precisely automate the wild blueberry harvester header

**DOI:** 10.1038/s41598-023-37087-z

**Published:** 2023-06-23

**Authors:** Zeeshan Haydar, Travis J. Esau, Aitazaz A. Farooque, Qamar U. Zaman, Patrick J. Hennessy, Kuljeet Singh, Farhat Abbas

**Affiliations:** 1 Faculty of Sustainable Design Engineering, University of Prince Edward Island, Charlottetown, PE Canada; 2 Department of Engineering, Faculty of Agriculture, Dalhousie University, Truro, NS Canada; 3 Canadian Center for Climate Change and Adaptation, University of Prince Edward Island, St. Peter’s, Canada; 4 College of Engineering Technology, University of Doha for Science and Technology, P.O. Box 24449, Doha, Qatar

**Keywords:** Mechanical engineering, Engineering

## Abstract

An operator of a wild blueberry harvester faces the fatigue of manually adjusting the height of the harvester’s head, considering spatial variations in plant height, fruit zone, and field topography affecting fruit yield. For stress-free harvesting of wild blueberries, a deep learning-supported machine vision control system has been developed to detect the fruit height and precisely auto-adjust the header picking teeth rake position. The OpenCV AI Kit (OAK-D) was used with YOLOv4-tiny deep learning model with code developed in Python to solve the challenge of matching fruit heights with the harvester’s head position. The system accuracy was statistically evaluated with R^2^ (coefficient of determination) and σ (standard deviation) measured on the difference in distances between the berries picking teeth and average fruit heights, which were 72, 43% and 2.1, 2.3 cm for the auto and manual head adjustment systems, respectively. This innovative system performed well in weed-free areas but requires further work to operate in weedy sections of the fields. Benefits of using this system include automated control of the harvester’s head to match the header picking rake height to the level of the fruit height while reducing the operator’s stress by creating safer working environments.

## Introduction

Lowbush blueberry (*Vaccinium angustifolium* Ait.) is one of the top revenue-generating commodities cultivated on a large scale in Atlantic Canada. In 2021, about 74,635 metric tons of blueberries were harvested from Canada's 69,016 ha area. The export value was 303 million CAD in 2021^1^. Blueberry fruit is very nutritious and has many health benefits, including dietary fiber, vitamin C, and antioxidants^2^; due to associated health benefits, various marketing campaigns increased the international demand for lowbush blueberries^3^.

Blueberry harvesting starts when 90% of the berries turn blue, and it begins after the first week of August and ends before the mid of September^4^. Traditionally, wild blueberries were harvested manually with a hand rake, which is very time-consuming and requires a substantial workforce to complete the task. The combined harvesting losses with the hand rake are 15–49%, which highly depend on and vary from person to person^5^.

The main challenges at the time of wild blueberry harvesting are short harvesting window^6^, labor shortage during the harvesting session^7^, high wage rate^8,9^, and over 43,000 ha field area of harvestable lowbush blueberry every year^10^. These issues motivated the growers toward mechanical harvesting, and investment in efficient harvesting and labor-saving technology can improve harvesting acreage^11^, reduce harvesting costs, and save time compared to less efficient methods like manual hand rake harvesting^10^. At the start of the harvester development, the header fruit-picking teeth hit the ground due to design limitations, which caused machine damage and fruit losses. Doug Bragg Enterprise (DBE) is a harvester manufacturer based in Nova Scotia. DBE improved the harvester header design by including a hydraulic control system to adjust the header according to the plant, the rotational speed with speed controller devices, and the harvester width to enhance machine efficiency^12^. The picking efficiency of the DBE harvester is 82–94% which depends heavily on the field conditions^4^. The latest DBE harvester works with the specification of 660.4 mm diameters, cylindrical picking header, and variable speed^13^. The harvesting closely relies on the operator's ability, and full automation would be a significant asset in detecting the plant characteristics and setting the header height^14^.

Various types of controller and feedback systems were tested with different agriculture machines to enhance productivity by reducing fruit loss, improving field efficiency, and for operator facilitation like feedback and control of many parameters in the combine harvesters^15^, semi-automated type combines harvesters^16^, on-the-spot analysis and feedback during the soil tillage operation^17^ moisture sensing and control of the irrigation valves^18^, fluid flow monitoring in spray machines^19^ and machine vision weeds detection system installed in wild blueberry harvester^20^. The modification by converting the wild blueberry small box-handling design into semi-automated handling increased the harvesting efficiency 18.5–22.7% and, reduced bin handling time by 55–68%^21^. Implementing fully autonomous wild blueberry mechanical harvesting, including auto steering and auto header harvesting control, offers significant benefits compared to total manual mechanical harvesting. This holds for expert and non-expert operators, as it effectively reduces heart rate by 13.83% and 19.3% respectively^22^.

A 20.3 cm hydraulic actuator was attached to the back of the header to quickly adjust the position off to ground according to berry height. Actuators are devices that work on the energy conversion principle and convert rotary motion into linear motion. The header-compatible electrical and hydraulic actuator was tested with the wild blueberry harvester, which took 13.96 s and 2.30 s respectively to fully stroke 20.32 cm of the head^14^. The electrical linear actuator accomplished the downward and upward speeds of 1.89 cm s^−1^ and 1.32 cm s^−1^, respectively, and the hydraulic actuator accomplished downward and upward speeds more quickly, 8.01 cm s^−1^ and 9.67 cm s^−1^, respectively^23^. Due to the quick response, the hydraulic actuator is a better and commercially suitable option for the wild blueberry harvester.

The operator is burdened with manually adjusting the header height, picking reel revolutions, and tractor steering, leading to a stressful job. However, the automation of header height control will significantly alleviate the operator's workload, simplifying their tasks and making them more manageable. Fruit yield, plant height, and ground slope can be assessed and mapped before harvesting using a ground-based multiple sensor system^24^. Georeferenced measurements of wild blueberry yield, ground slope, and plant height can be obtained by capturing images, collecting slope sensor signals, and recording ultrasonic voltage signals using a combination of real-time kinematic global positioning system^25^. However, to achieve real-time header adjustment, we require an on-site detection and decision system capable of accurately and promptly adjusting the positioning of the harvester header. Artificial Intelligence (AI) plays a vital role in agriculture and is very helpful for timely disease detection, scouting, and management^26^. Machine vision technology detects weeds and plants based on their texture and morphological features^27,28^. The mathematically based model, like the artificial neural network (ANNs), works like the human brain neural network. The Deep learning convolutional neural network (DCNNs) is an upgraded version of ANNs and can extract complicated features from the object image with high precision^29^.

Among the deep learning model, the You Only Look Once (YOLO) frameworks are widely used in various applications for object detection, excellent performance in latency, and provide the tradeoff between accuracy and speed, which significantly help to deal with different scenario^30,31^. YOLOv4 is used for numerous applications in the agriculture field. It can detect the multiple stages of the cheery for accurate picking for more economical harvesting^32^ and accurately recognizes the occluded objects^33^. The YOLOv4-tiny is faster as compared to YOLOv4 and perform better in real-time detection in term of time and computational cost^34,35^, fast single detection mode, and precision even at high speed^36^. In electronics industries, YOLOv4-tiny can detect small-size electronic components by maintaining high accuracy at a faster speed^37^. The YOLOv4-Tiny neural network model performed better among the YOLOv3, YOLOv3-Tiny, YOLOv3-SPP, YOLOv4, YOLOv4-Small for the detection of wild blueberry maturity stage in term of computation load and the interface time is 7.8 ms for a single 1280 × 736 resolution image data^38^.

The stereo vision depth camera modules can capture the targeted object's color image and depth information using the triangulation concept from baseline by comparing the matching pixels of images from different cameras. These cameras are used in various applications like the Kinect RGBD camera used to determine the maize plant height^39^ and Kinect V2.0 depth for the green paper plant height^40^. The E450 Olympus with the segmentation algorithm for accessing the maize height^41^ and GO-5000 active pixel sensor for the wheat plant height measurement^42^. Intel real sense D435 for the basil plant height in pots^43^, Tara USB 3.0 with the tilt fusion angle for plant height measurement^44^. Using stereo vision camera technology with different algorithms is a decent approach to knowing plant features for growth monitoring, analysis, and decision-making^45^.

The main goal of this study is to automate the harvester header teeth rake height setting on the go using 3D camera vision technology. This research will be a vital step forward in rapidly operating the harvester and reducing the workload stress of the driver to adjust the header frequently. This development will increase harvesting efficiency, save time, and reduce operator fatigue. The results and findings from this project will improve understating for future research work in using camera vision technology for header automation.

## Materials and methods

### Images dataset and CNN model training

Convolutional Neural Networks (CNNs) were trained using the 6766 images dataset collected for the fruit maturity stage study^38^. The image data were collected from 54 points of each four fields to cover the whole area with significant clonal variation in each wild blueberry field^46,47^. This was done to ensure that the dataset captured the range of variation in the wild blueberries across the fields to train a more robust model. Empirical studies have demonstrated that the optimal approach involves allocating 20–30% of the data for testing purposes while utilizing the remaining 70–80% for training the model^48^. In this study, 70% of the images (4736) were used for model training, 15% (1015) for validation, and 15% (1015) for testing to prevent the model from overfitting and accurately evaluating.

The collected raw image data set size was 1280 × 720. To find the best resolution size to get maximum model accuracy, the YOLOv4-tiny model was trained at three different image resolution sizes (i) 416 × 416, (ii) 608 × 608, and (iii) 832 × 832 to figure out at what images size model provide the maximum mean average precision (mAP). The model was trained to a batch size of 64 with subdivisions of 16. The maximum batch taken was 4000. Steps taken for the training were 3200 and 3600, respectively (80% and 90% of the maximum batch). The YOLOv4-tiny model was trained on Google Colab GPU, and VRAM usage was about 1.87 GB at image size 832 × 832. Ultimately, the model weight file was converted into the MyriadX Binary Large Object format (BLOB) to optimize the best interference with the visual processing unit and allow the depth AI module to use a customized trained model.

### Experiment field

**Figure 1:**
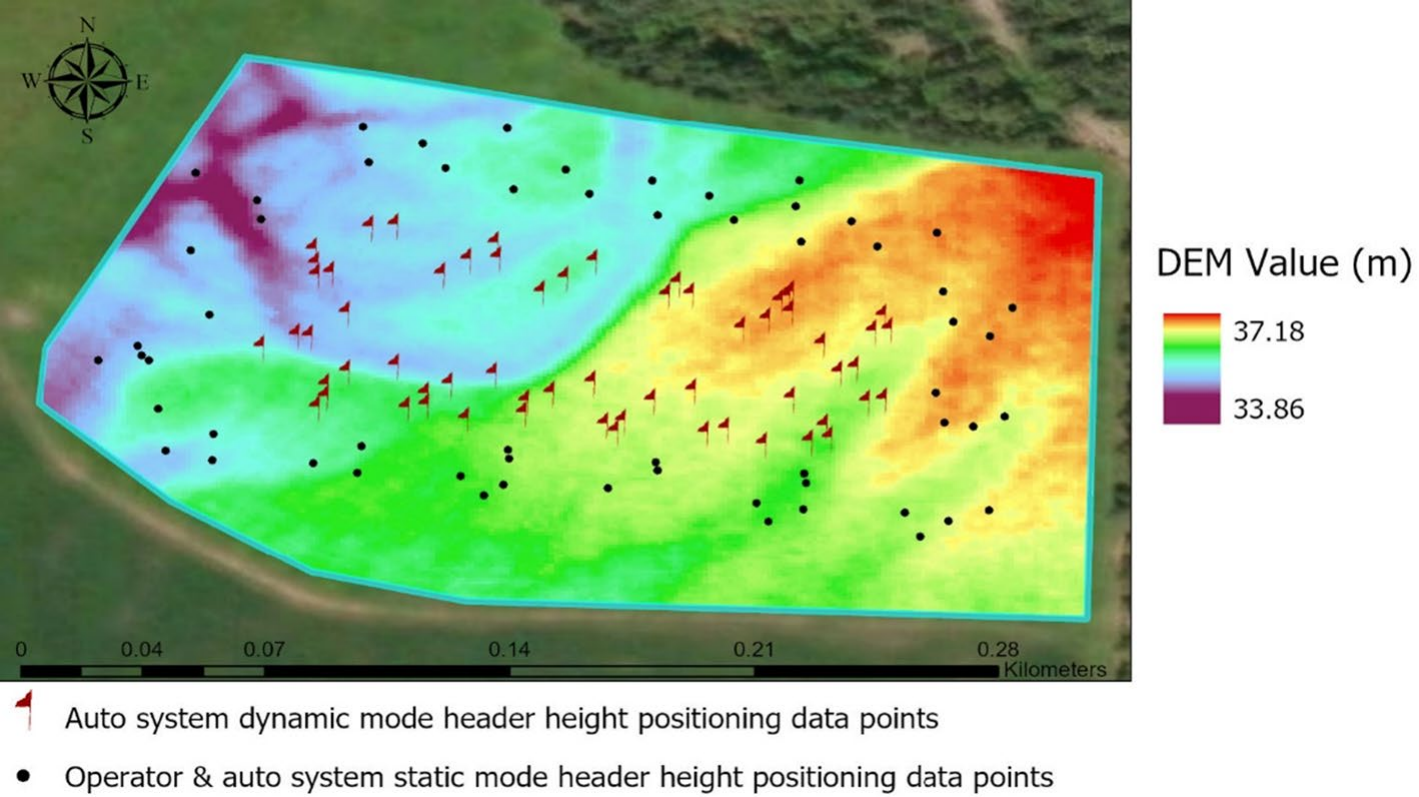
Boundary map of the experimental field and data collection points distribution, Image source: Esri, Maxar, Earthstar geographics, and the GIS use community.

The developed system was evaluated in the McCully field (45.42468827° N, 63.473859° W), Debert, Nova Scotia, shown in Fig. 1. Among the total of one hundred twenty location points, 60 points were used for static measurement and the other 60 for the dynamic auto header height adjustment system scenario. The study field area was rectangular, and the wild blueberry harvester header was attached to the left side of the tractor. For optimum picking performance, harvesting each side of the field is preferred while traveling in a straight line. The data point locations were marked using a true random number technique to determine field plots^49^ using an online random number generator. At each field-side after the first random point from the corner, the next points were marked at a fixed 20-m distance until reaching the next field corner. This procedure was repeated at each corner to ensure that the sample represented the overall field to avoid bias. The automatic header height positioning system was evaluated under two different operating conditions (i) Static mode: the automatic system was used to adjust the header height while the harvester was stationary at a marked field location by switching from manual to auto system (ii) Dynamic mode, the automatic system was used continuously during harvesting without any manual intervention, and measurements were taken when the harvester reached each marked location in the field.

In Fig. 1, the black dots are the locations where the operator's manual harvester header height adjustment was recorded, and the static mode of the automatic height adjustment system was evaluated. The red flags show locations, where the dynamic scenario of harvesting the automated header height adjustment system was assessed.

### Development of a control system

**Figure 2:**
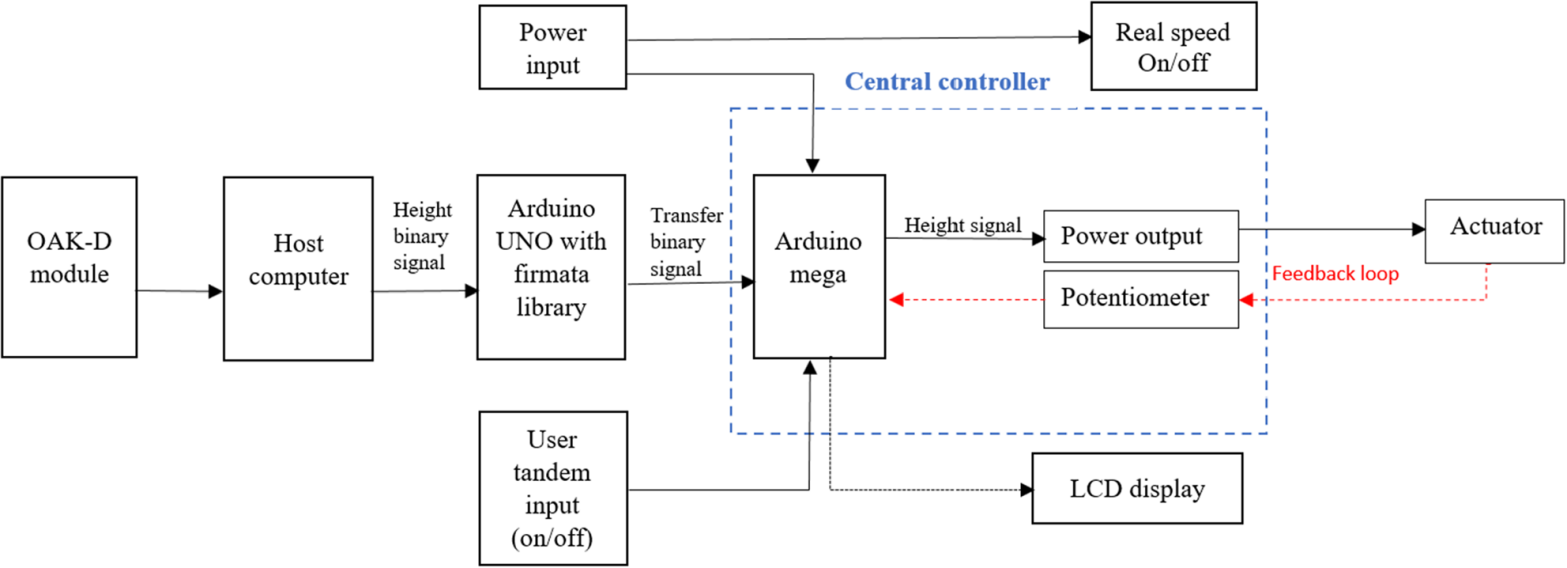
Communication block diagram of automatic harvester header height positioning system.

**Figure 3:**
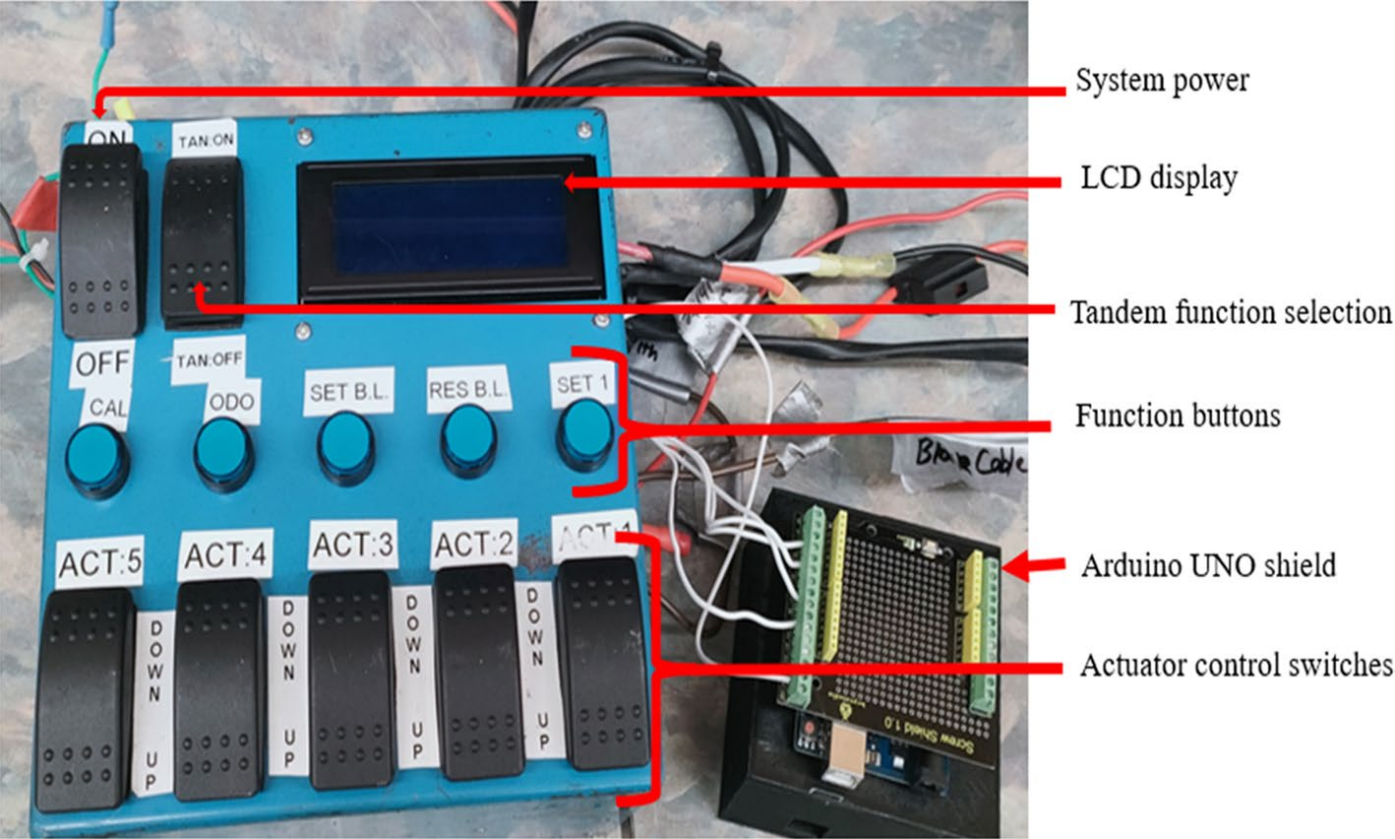
Control functions of custom build harvester header control box.

For the automatic header picking height adjustment based on the berry height, a machine vision system using the OAK-D camera module and deep learning was integrated into the developed harvester head controller^14^. After modification, the already-developed controller worked as a central controller in the new system. The new system featured a closed loop feed control system, as explained in block diagram Fig. 2, consisting of one Luxonis Spatial DepthAI OpenCV module OAK-D, a host computer (ThinkPad L14 with 16 GB RAM), a Linear potentiometer (LPPS-22 series), header height positioning central controller, and one Arduino UNO for serial communication as shown in Fig. 3.

The central control takes the continuous values in integer format from the host computer through serial communication and from the linear potentiometer to match both readings. The three-position momentary rocker switch moves the actuator up and down to compensate for the difference. Suppose the current height is less than the recommended height by the host computer; in that case, the controller sends the signal to the mechanical relay to change the header position and moves up to the actuator stroke using the hydraulic power control and vice versa. A customized spatial detection Python library (https://github.com/luxonis/depthai) of DepthAI was continuously run in the host computer to calculate the wild blueberry height, and a Pyfirmata library was included in the Python code to send the calculated header-picking height signal to the controller through the Arduino UNO to adjust the header position.

The OAK-D Module is driven by the Intel Myriad X VPU (vision processing unit) and powered by USB type-C from the host computer. Its baseboard comprises the two 720 pixels/120 Hertz global shutters in synchronized stereo pairs and one autofocus color 12 MP/4 K rolling shutter camera. The two mono cameras are used to create a disparity map which can be used to calculate the distance, and the color camera is for digital zoom, better color representation, and texture information. The Intel Movidius Myriad X technology makes it possible to run the deep neural network on the device and output data to the host device via USB 3.1.

**Figure 4:**
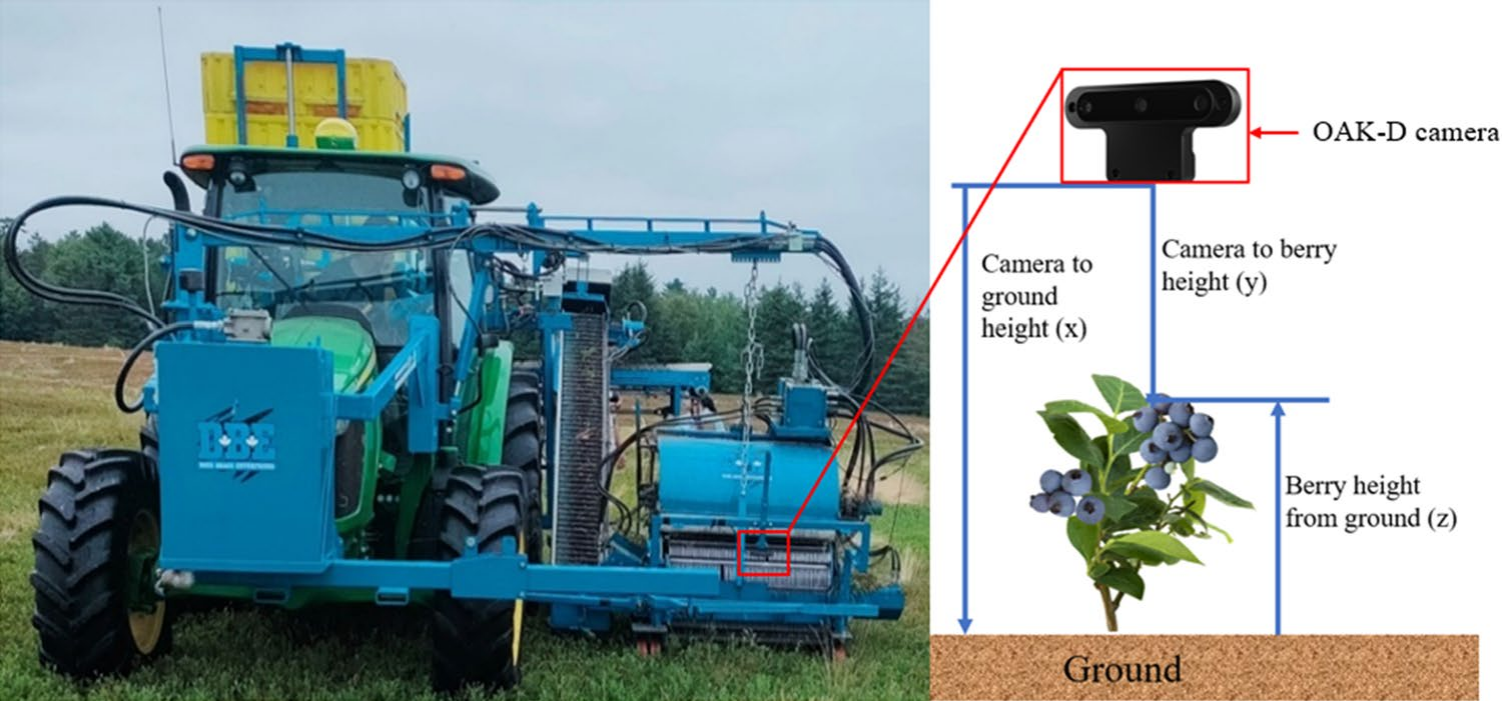
The left side is the wild blueberry harvester with a camera mount in front of the header, and the right side of the images shows how the camera to berry height (y) is used to calculate berry height from the ground (z) using Python code.

The OAK-D was set up in front of the harvester header by attaching an extra assembly bar with the height adjustable mount as shown in Fig. 4. The camera position is set downward to detect the wild blueberries from the plants. The Lenovo ThinkPad L14 with 16 GB RAM and Windows 10 was set up as a host computer within the tractor cabin. The laptop directly powered the camera via a USB port and connected using a specific 5-m-long USB 3.1 cable.

To get the position status of the actuator in real-time, a 25.4 cm linear potentiometer (LPPS-22) was installed with the actuator on the back side of the harvester header as a feedback sensor. The potentiometer is precise and has self-aligning swivel rod eyes on both ends, which makes it very easy to connect with the actuator without any significant modification. This sensor inner rod was 5.08 cm longer than the original actuator stroke length of the harvester, which was taken as a base value and considered in all the calculations. This feedback sensor was directly attached to the central control to give the value of the header position.

### Auto berry height measurement

Measuring the accurate blueberry height from the ground is essential for positioning the header. For this purpose, the spatial detection Python code was customized by adding mathematical Eqs. (1) and (2) to filter the height data while removing the noise in data, the berry height calculation formula, and the header height corrections pipelines. In the field on the go, the camera module detects many blueberries in the camera frame of view (FOV) range, but the header positioning system needs a single value of the berry distance from the ground. The rolling average of the nine berries’ distance from the camera was used to calculate the final average berry height from the ground using the following Eq. (1), as explained in Fig. 4.

1 $${\text{z}} = {\text{ x}} + {\text{ y}} - {\text{h,}}$$

where ‘h’ is the height correction value obtained from Eq. (2).

When the tractor turns or due to sudden bumps in the field, the camera detects berries that are not directly under the camera. These values are high to the distance value fixed for the camera to the ground, considered an outlier, and not included in the calculation. The initial plant and berry height collected data helped to set the filter range. Lines of codes were added to the Python code to filter out the values 45 ≤ Z-value ≥ 75 cm, so by this filtering, the code only includes those values within the range and takes the average when it detects the nine berries.

While harvesting the height of the header picking unit change according to berry height, with that, the camera height from the ground is also changed, which we initially fixed as a base height. To find the real-time difference in camera height for precise measurement OAK-D camera built-in Inertial Measurement Unit (IMU) BNO085 sensor values were used. This IMU sensor is 9-axis which can record the accelerometer, gyroscope, and magnetometer values. The accelerometer can measure the rotation around all three (X, Y, Z) axes and calculate pitch, yaw, and roll values. The IMU sensor's built-in position on the module is like when the camera is fixed and pointed downward; the rotation only comes around the y-axis, called pitch. The IMU pipeline was included in the Python coding to correct the change in camera height in real-time.

A real-time relationship was developed using linear regression to calculate the correct camera height from the pitch value. The workshop's initial trial was done by installing the camera on the mount. The initial camera height was 75 cm from the flat ground surface. The camera was mounted in a way that as the header moves up, the distance between header teeth and the ground increases, but the camera moves downward, and its height decreases. The pitch value changed between 5° to 16° values at the minimum and maximum header height positions, and in the field, it usually varies from 9° to 14°. The camera heights from the ground were measured using the scale at every pitch change. The difference between the initial height and height by changing the header position was calculated, and the relationship was developed using a linear regression method.

2 $${\text{h }} = \, - 0.{\text{76d }} + { 11}.{68}{\text{.}}$$

The code used the developed equation to find the change in the camera to ground distance. Here d is the pitch value in degree, and h is the height correction value (cm) which is the change in initial camera height from the ground. The correction value was subtracted from the initial fixed camera height for accurate berry height measurement, as illustrated in Eq. 1. The camera height was noted using a one-meter stick to compare the generated auto-correction value and the difference in the camera’s initial and present height at each data point location.

### Berry fruit height measurement

**Figure 5:**
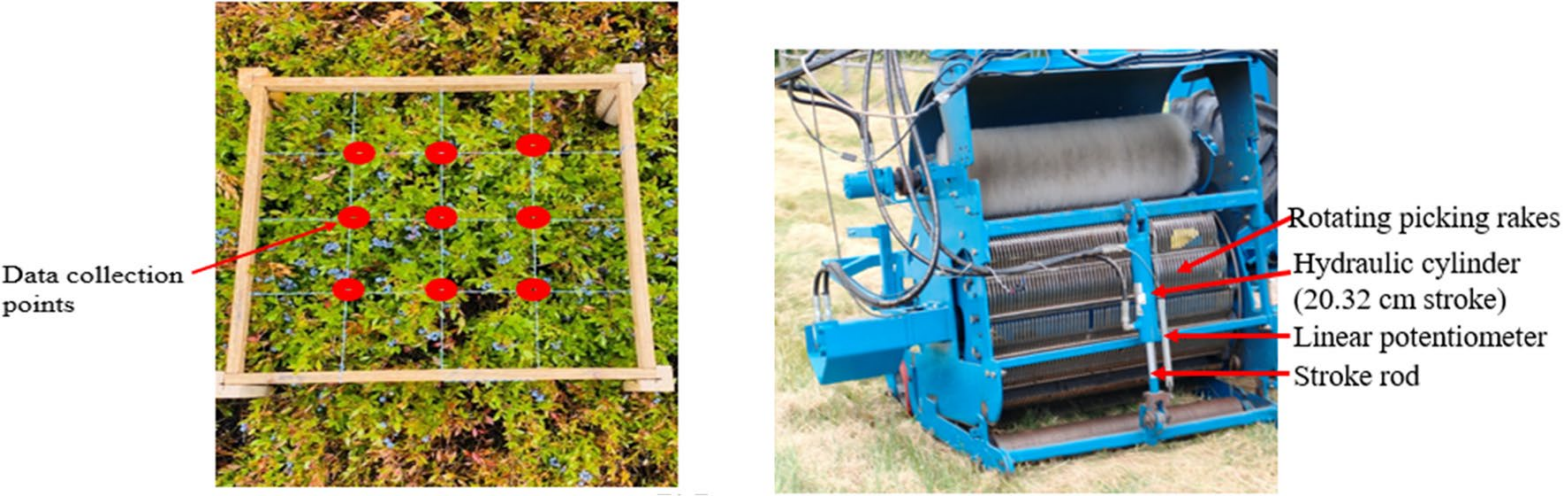
(**a**) Fruit height data collection quadrant. (**b**) Wild blueberry harvester header.

The plant's lowest and highest berry heights were measured at each marked location using a ruler to evaluate the performance of the auto system. For this purpose, a specific quadrant (41 cm × 41 cm) shown in Fig. 5a was used to follow the same procedure to measure the berry height throughout the field. The lowest and highest berry height at plants were measured at the intersection of wires or from the plant close to the intersection in case any plant is not at the intersection. This measurement helped to compare and understand the variation in the fruit zone and the setting of the harvester header picking teeth.

### Manual stroke height measurement

The open stroke length was measured using a ruler to find the harvester header picking teeth height from the ground, as shown in Fig. 5b. The driver sets the stroke height based on the plant height, weeds, and slope by visualization from the tractor cabin, while the auto height adjustment system takes the nine berries height and pitch value for camera height correction to set the header height. For comparison, the stroke readings were noted separately at the marked points for both operator and auto systems.

### Statement: data collection from wild blueberry fields

Necessary permissions were obtained from the grower to collect the plant height data and to check the performance of the harvester header positioning system in field. In this study, we comply with the IUCN policy statement on research involving species at risk of extinction and the convention on the trade in endangered species of wild fauna and flora.

## Results and discussion

### Convolutional neural network training

**Figure 6:**
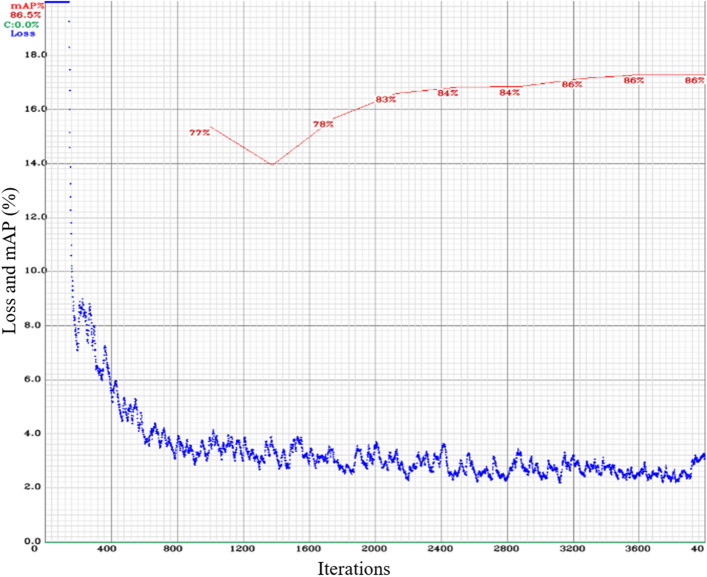
The performance parameter Mean Average Precision and Loss of YOLOv4-tiny model training at image size 832 × 832 pixels.

When choosing trained models, the Mean Average Precision (mAP) and loss are important factors. Among them, the YOLOv4-tiny model is the preferred option because it offers a fast and efficient detection system with a minimal interference time of only 0.7 ms. The convolutional neural network accuracy depends on the image size, and it impacts architecture parameters like information loss, training parameters, and model complexity^50^. The YOLOv4-tiny model trained an image size 832 × 832, resulting in an mAP of 86.5% and a loss value of 3.28, as shown in Fig. 6. For image sizes 416 × 416 and 608 × 608, the mAP values were 76% and 83.4%, respectively, while the corresponding loss values were 1.26 and 2.75. These metrics provide insights into the model's performance on different image sizes.

In the case of wild blueberry plants, the fruits tend to grow closer together on the plant branches, and the plants themselves may be tilted in various directions. Therefore, to automatically adjust the harvester header, precision becomes crucial. The primary objective of the model training is to locate the berries accurately rather than measure their specific characteristics. To evaluate the entire system in real-field scenarios, the trained model's binary large object (blob) file was integrated with the OAK-D camera, generated at an image size of 832 × 832. This integration allows for a comprehensive assessment of the system's performance.

### Berry height comparison

The accurate measurement of berry height is of utmost importance in real-time to improve picking efficiency. The study findings *p* = 0.230, greater than 0.05, indicating no statistically significant difference in the average berry height measured by the manual method and the camera vision auto system. The same plant's lowest and highest berry height data were collected during the manual measurement. In contrast, the camera vision system detected random berries within that section. Upon calculating nine values, the system determined the average height and transmitted this information to the controller to adjust the header. Table 1 illustrates that the standard deviation is relatively high in the camera vision system, suggesting that the camera predominantly detects the top and middle fruits of the plant. As a result, the control system performed final adjustments to optimize the harvesting process, thereby evaluating the system's overall performance, as depicted in Table 2.

**Table 1:** Fruit height measurement comparison.

Sample	N	Mean	St Dev	SE Mean
Manually measured fruit height (cm)	60	13.12	2.95	0.38
The camera measured fruit height (cm)	60	12.45	4.81	0.62

**Table 2:** Paired t-test results of camera height change versus height correction.

Sample	N	Mean	St Dev	SE Mean
Camera height difference (cm)	60	0.86	2.01	0.26
Height correction (cm)	60	0.23	1.82	0.23

### Camera height correction

During the harvesting by the automatic header height positioning system, the position of the header changed frequently based on the berry height. So, the initially fixed camera height changes for every new position. The test results are shown in Table 2 to find the relationship between the correction value generated by the developed equation and the difference between the initial set and the current camera height from the ground at data points marked locations. The results *p* = 0.022 indicate a significant difference in correction value and the change in camera height. However, the other parameters, such as standard deviation and standard error, show that the relation can be applied because of the same range of variation in value. The difference is due to the high vibration on the harvester header during harvesting and the inclination angle in the header because of the semi-mounted type and field slope.

### Header teeth rake height positioning

A complete control system was set up on the tractor to test and evaluate the performance of the auto header height system to position the teeth height from the ground. The teeth height from the ground is half the open stroke length of the hydraulic actuator. The operator manually adjusts the header while harvesting based on visualization from the cabin until the arrival at the marked location and stops the tractor without disturbing the header position. The open stroke lengths were measured using the scale. The second measurement was taken at the same spot to evaluate the auto header height positioning on the static mode by switching the manual system to an auto header positing system using the tandem function button on the central controller, as shown in Fig. 3. The camera detected the berries and sent output to the host computer, which the controller processed to set the header height accordingly. In the static mode, the linear regression R^2^ resulting is 0.85, indicating a significant linear relationship between the camera-measured berry height and the automatically set header teeth rack height from the ground.

**Figure 7:**
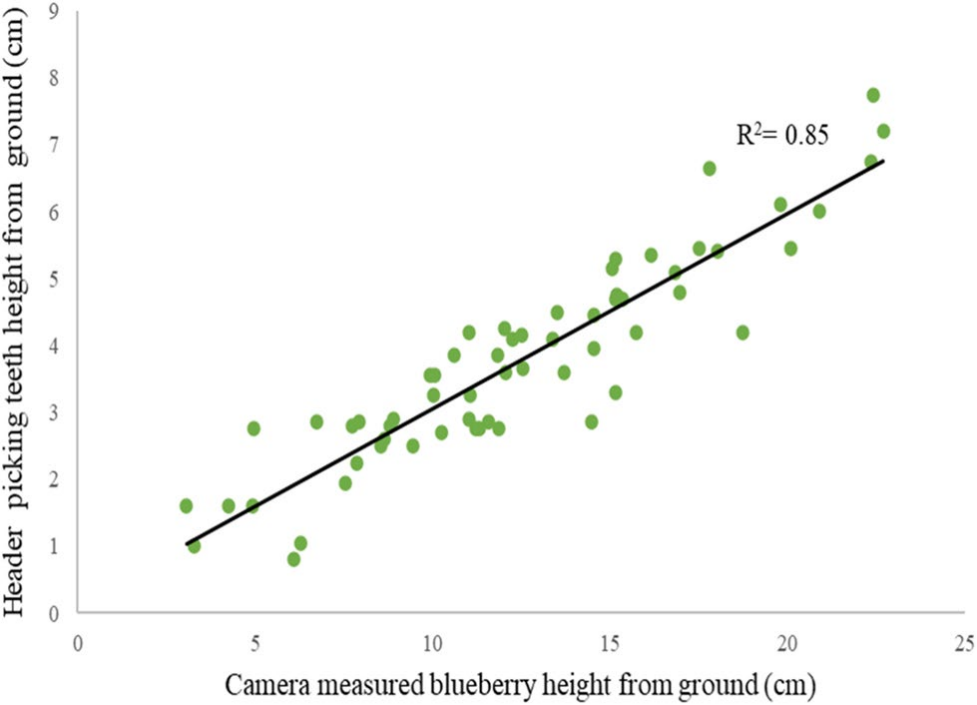
Header height picking teeth positioning based on wild blueberry height.

After trials, training, and successfully running the system in static mode, as shown in Fig. 7, the system was tested for the dynamic scenario. The harvesting was carried out entirely using the automatic header height positioning system without manual intervention on the other sixty marked locations.

**Figure 8:**
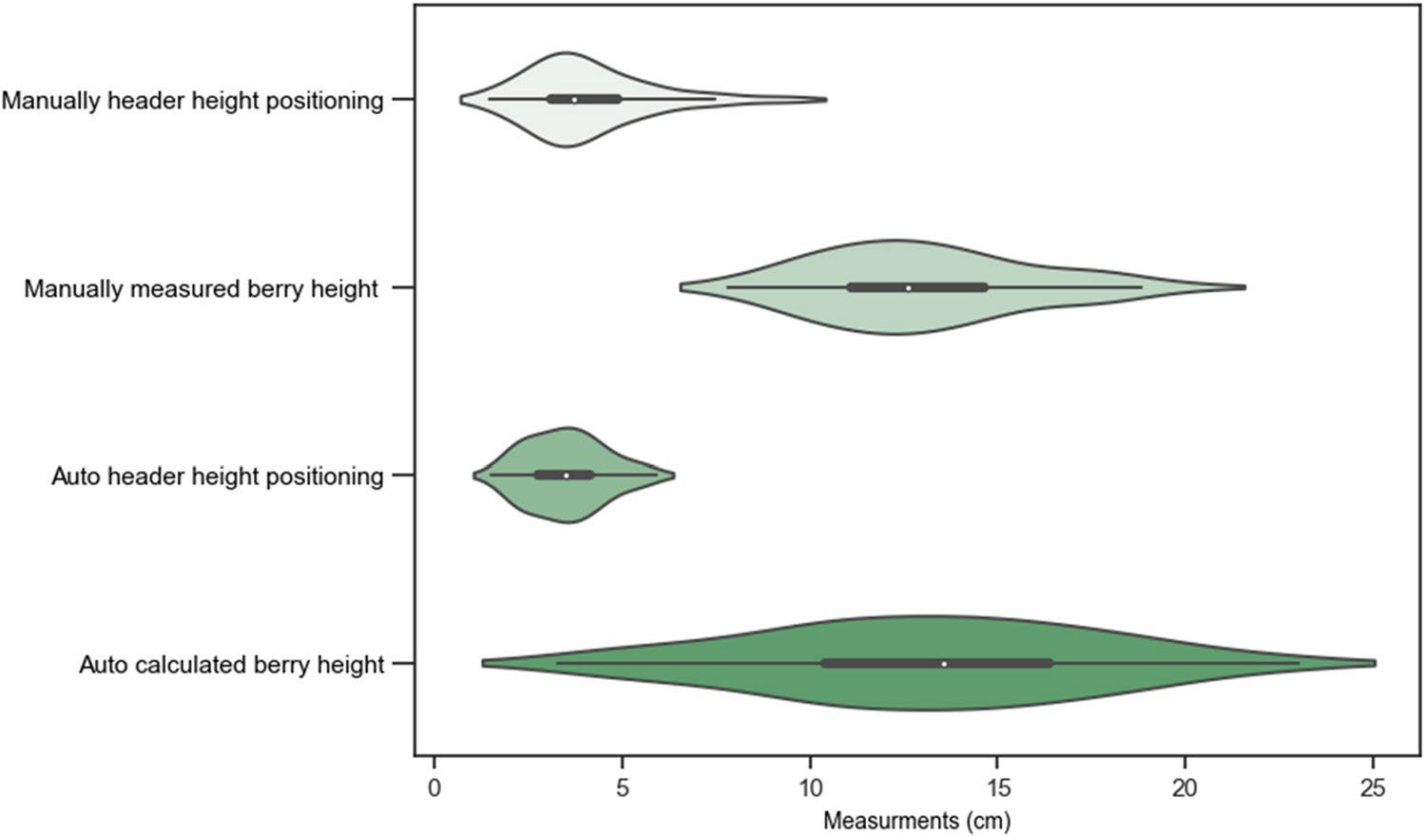
Visualizing the distribution of measured plant and header height from ground.

**Figure 9:**
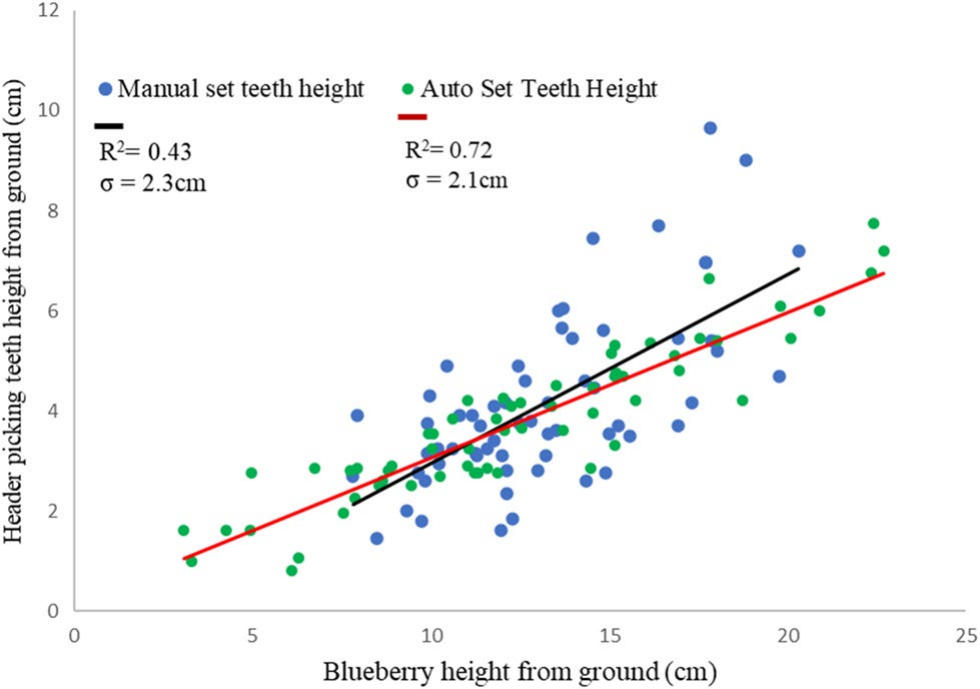
Comparison of the auto system and operator adjusted header teeth rake positioning results.

As shown in Fig. 8, a violin plot visualizes the probability distribution of all variables. The white point in the violin plot inside the box is the median value of header teeth rake height from the ground, which is 3.7 and 3.5 cm for the manual by the operator and auto header height positioning, respectively. The results show that the automatic system can adjust the header height similarly to the operator. Sometimes, the operator raises the header height to avoid debris, particularly in the weed zone. In contrast, the camera vision system didn’t consider it because the model was trained for fruit detection only. That’s why the maximum value of the manual header height positioning is high than auto header positioning. The wild blueberry height in this field varies from 7 to 23 cm. Figure 8 shows that the automatically calculated berry height using the camera can accurately measure berry height across a range of values. The relationship between berry height and harvester header teeth rake height for both cases is shown in Fig. 9.

The header teeth rake height from the ground set by the automatic positioning system (R^2^ = 0.72) is more rational to the trend line than the setting done by the operator (R^2^ = 0.43). The average blueberry height was used for the comparison because the plant's lowest and highest berry heights throughout the quadrant could not be constant.

Furthermore, the standard deviation between the height difference between the blueberry and teeth was calculated for both cases of header positioning by the operator and auto system, which are 2.3 and 2.1 cm, respectively. As the standard deviation is low, the teeth will be closer to the blueberry and off to the ground and vice versa. In both cases, the setting of the header teeth rakes and blueberry picking is fulfilled, but statistically, teeth setting by the auto system yields more significant results.

In areas designated as weed-free zones, the effectiveness of the auto header height adjustment positioning system is significantly enhanced due to its ability to detect wild blueberry fruits accurately. The system ensures precise header positioning based on the height of the fruit. However, challenges arise when the fruit is concealed by weeds, making it difficult for the system to identify the presence of fruit promptly. In such cases, the system relies on its previous header position until it detects the next nine wild blueberries successfully. The operator sets header height based on visual observation like plant height, weeds, and slope but not on any calculation, so sometimes the operator header setting is good and sometimes not as it should be. Using the deep learning model, auto-based teeth adjust the header teeth rake height more precisely based on berry height.

## Conclusion

During wild blueberry harvesting, the operator must pay close attention to keeping the header height at the appropriate position throughout the field according to the berry height. The machine vision control system using deep learning for berry detection proved to be a suitable approach for automatically setting the header teeth rake for efficient picking and reducing the workload stress on the operator. To set the picking teeth rake position, the operator considers the berry height, slope, fruit zone, fruit yield, and weed density, while the auto system takes the berry height as an input for the picking teeth positioning. The auto header height positioning system will allow the operator to give attention to other tasks like controlling the tractor steering to keep the line straight for efficient overlapping from the passed lap and fruit bin handling. This technology could help to manage the individual adjustment on the multi-header machine and help to increase the field efficiency and reduce energy consumption. As technology is evolving in the convolutional neural network and camera stereo vision technology with time, so more fine results can be obtained by training with advanced neural networks like YOLOv7, and the stereo vision module has a global shutter color and fixed focus camera because the autofocus camera at high field bumps blurs the video frame and take some time to readjust.

This study demonstrated the successful deep learning supported machine vision control system to precisely auto-position the header teeth rake of a mechanical wild blueberry harvester concerning berry height with R^2^ = 0.72 and a standard deviation of 2.1 cm of the height difference between the berry and teeth rake. In the field where the weed patch is dense, it becomes difficult for an automated header height positioning system to detect the location of the berries due to the long and high weed density. So, the system keeps the harvester at the previous header setting until it again detects the next nine berries for a rolling average; in the manual adjustment operator keeps the header position high in a dense weed area to reduce the debris on the conveyor belt with blueberries. In weed-free zone areas, the automated header height positioning system can set the header similarly or even better than the operators. Furthermore, the results of this study represent encouraging feedback on the realization of a fully automated harvester.

## Data Availability

Data can be provided on request to the corresponding author.
